# Soluble programmed death molecule 1 (sPD-1) as a predictor of interstitial lung disease in rheumatoid arthritis

**DOI:** 10.1186/s12865-021-00460-6

**Published:** 2021-10-15

**Authors:** Li Xu, Lichun Jiang, Liuyan Nie, Songzhao Zhang, Lei Liu, Yan Du, Jing Xue

**Affiliations:** 1 Department of Rheumatology, The Second Affiliated Hospital of Zhejiang University School of Medicine, Hangzhou, 310009 China; 2 Department of Medical, Jiaxing University Affiliated Women and Children Hospital (Jiaxing Maternity and Child Health Care Hospital), Jiaxing, 314000 China; 3 Department of Clinical Laboratory, The Second Affiliated Hospital of Zhejiang University School of Medicine, Hangzhou, 310009 China; 4 Department of Rheumatology, Jiaxing First Hospital, Jiaxing, 314000 China

**Keywords:** sPD-1, Rheumatoid arthritis, Interstitial lung disease (ILD)

## Abstract

**Background:**

Previous studies have indicated that the programmed death molecule 1 (PD-1) signaling pathway may play a key role in rheumatoid arthritis (RA). However, the pathogenesis of rheumatoid arthritis-related interstitial lung disease (RA-ILD) is not clear. We examined the serum levels of soluble PD-1 in patients with RA and its relationship with RA-ILD.

**Methods:**

Blood samples were obtained from 87 patients with RA (58 with ILD and 29 without ILD) and 45 healthy controls. Serum sPD-1 was measured by Enzyme-Linked Immunosorbent Assay. The pulmonary interstitial disease score was completed by a pulmonary physician and a radiologist through chest high-resolution computed tomography. Patients with RA-ILD were tested for lung function [e.g., forced vital capacity (FVC%), diffusing capacity of lungs for carbon monoxide (DLCO%)]. Associations between ILD and various markers, including sPD-1 and confounding factors, were investigated by logistic regression analysis. Diagnostic values of sPD-1 for the presence of ILD were investigated using receiver operating characteristic curve analysis.

**Results:**

Serum sPD-1 levels were higher in RA patients with ILD than in RA patients without ILD and healthy controls (185.1 ± 109.0 pg/ml vs. 119.1 ± 77.5 pg/ml vs. 52.1 ± 21.7 pg/ml, *P* < 0.05). Serum sPD-1 levels were positively correlated with RF titer (*P* = 0.02, r = 0.249), anti-cyclic citrullinated peptide antibody status (*P* = 0.02, r = 0.243), and serum IgG levels (*P* < 0.001, r = 0.368), negatively associated with FVC% (*P* = 0.02, r = − 0.344), forced expiratory volume (FEV1%) (*P*  = 0.01, r = − 0.354), total lung capacity (TLC%) (*P* = 0.046, r = − 0.302), and was independently associated with the presence of ILD in RA patients by multivariate logistic regression analysis. The sensitivity and specificity of sPD-1 levels for the detection of ILD in RA patients were 58.6% and 75.9%, respectively. The area under the curve was 0.689.

**Conclusion:**

Serum sPD-1 levels were increased in RA patients with ILD. Increased sPD-1 may be a valuable biomarker to predict the presence of ILD in patients with RA.

**Supplementary Information:**

The online version contains supplementary material available at 10.1186/s12865-021-00460-6.

## Background

Rheumatoid arthritis (RA) is a chronic and complex autoimmune disease that causes inflammation and bone destruction in joint areas. It is characterized by a systemic inflammatory state that mainly affects joints but also other organs, such as skin, eyes, lungs, and the cardiovascular system. Early diagnosis and management of interstitial lung disease (ILD) in RA patients remains a big challenge.

In recent years, rheumatoid arthritis-related interstitial lung disease (RA-ILD) has been an increasingly recognized disease. It is the main cause of death in rheumatoid arthritis (RA) patients. Epidemiological studies have reported that the mortality of RA-ILD is about 6–13% [1, 2]. The etiology of RA-ILD is still unclear, but may be related to smoking, oxidative stress, and other factors that activate autoimmunity and the attack of post-transcriptionally modified self-proteins, such as citrullinated peptides. This phenomenon usually occurs in the synovial tissue of joints. Citrullinated peptide can also be produced in the lungs of some patients, causing a similar immune response and lung fibroblasts to be activated and differentiated into myofibroblasts, leading to pulmonary fibrosis [3]. Therefore, the early identification of RA-ILD in patients is critical.

Programmed cell death-1 (PD-1) is a major immune checkpoint molecule implicated in immune-suppression and immune-tolerance. It is expressed in activated T cells as well as activated B cells, NK cells, and monocytes [4]. PD-1 has two ligands, PD-L1 and PD-L2, which combine to transmit inhibitory signals, participate in human cellular immunity and humoral immunity, and play a negative regulatory role in the immune response [5]. Anti-PD-1/PD-L1 therapeutic antibodies have achieved great success in the area of oncology. There is compelling evidence from experimental mouse models, as well as from clinical studies in humans, that the PD-1 signaling pathway is involved in the pathogenesis of various chronic inflammatory and rheumatoid arthritis diseases [6, 7]. These studies indicate that the PD-1 signaling pathway plays a key role in the occurrence and development of RA. At present, the relationship between PD-1 and RA-ILD has not been reported. PD-1 has membrane and soluble forms, and the soluble form is easier to measure. In the present study, we aimed to examine the expression levels of serum sPD-1 in patients with RA and investigate its relationship with RA-ILD.

## Methods

### Study population

Fifty-eight random rheumatoid arthritis patients with ILD were recruited for the RA-ILD group. Twenty-nine random RA patients were picked for the RA group. All patients were recruited from the Second Affiliated Hospital of Zhejiang University School of Medicine. Forty-five healthy controls were recruited from nearby communities. Cases and controls were of similar age and had a similar sex distribution. The inclusion criteria were as follows: (1) Diagnosis of RA according to the Guideline of the American College of Rheumatology classification of RA from 1987; (2) patients aged ≥ 18 years. Exclusion criteria included a history of, or any current, symptomatic or uncontrolled heart, lung, and kidney disease, active infection, malignant tumors or other systemic autoimmune diseases.

Peripheral blood was collected from all patients, then serum was isolated and stored at − 80 °C until further use in our laboratory. Samples were collected from December 2019 to February 2021. The clinical data, laboratory indexes, and imaging data from the patients were recorded, and included sex, age, course of the disease, respiratory-related manifestations, smoking history, the treatment (glucocorticoid and immunosuppressive drug therapy), number of swollen joints, number of tender joints, anti-cyclic citrullinated peptide (CCP) antibody, rheumatoid factor (RF), c-reactive protein (CRP), erythrocyte sedimentation rate (ESR), high-resolution computed tomography (HRCT) score, pulmonary function [forced vital capacity (FVC), carbon monoxide diffusion rate (DLCO)] and other related examination results. More details of each patient’s characteristics are shown in Additional file 1: Tables S1 and S2.

Our study was approved by the ethics committee of the Second Affiliated Hospital of Zhejiang University School of Medicine.

### Clinical assessment of patients and ILD diagnosis

The diagnostic criteria for RA-ILD followed the clinical diagnostic criteria for idiopathic pulmonary fibrosis (IPF) proposed by the American Thoracic Society and European Respiratory Society in 2002 [8]. Inclusion criteria were:

1. *Clinical symptoms* dry cough, chest tightness after exercise, shortness of breath, cyanosis, Velcro rale, and clubbing finger;
2. *Pulmonary function examination* mainly restricted ventilation dysfunction and decreased diffusion function, forced vital capacity (FVC) < 80%, diffusing capacity of the lungs for carbon monoxide diffusion (DLCO) < 80%;
3. *Pulmonary HRCT fibrosis* irregular linear shadow, grid shadow, thin-wall cystic change, honeycomb change, ground glass density, shadow, bronchial vascular bundle thickening or pulmonary bullae, bronchiectasis, interlobular septal thickening, and subpleural nodule focus.

### HRCT score

There were three specified layers of HRCT scan included in the total score: the upper margin of the aortic arch, the carina, and 1 cm above the diaphragm [9]. The percentages of six layers of fibrosis in the corresponding lung field area were calculated and scored according to the lesion involvement area (Table 1). An overall CT score was obtained by adding the bilateral six averaged scores assigned by two independent radiologists.

**Table 1:** Quantitative criteria for lung tissue involvement

Score	HRCT performance
0	NA
1	1–25% involvement
2	26–50% involvement
3	51–75% involvement
4	Range greater than 75%

*HRCT* high-resolution computerized tomography

### Detection of sPD-1 by enzyme linked immunosorbent assay (ELISA)

The ELISA kit for sPD-1 was purchased from Invitrogen (American). The concentration of sPD-1 was measured according to the manufacturer's instructions. Briefly, recombinant human PD-1 standard was reconstituted using distilled water to a concentration of 300 pg/mL, and diluted to provide a concentration range from 150 to 2.34 pg/ml. Samples and standards were added to ELISA plate wells as per the manufacturer’s instructions, and sample diluent alone was added to blank control wells. Diluted biotin-conjugate was then added to each well, mixed and incubated for 2 h at room temperature, followed by four washes with wash buffer. Diluted streptavidin-HRP was then added to each well, mixed and incubated for 1 h at room temperature, followed by four washes with wash buffer. Supplied TMB substrate solution was added to all wells, mixed and incubated for 30 min at room temperature in the dark. Then, supplied stop solution was added and the absorbance (to calculate optimal density and then sPD-1 concentration) of standards and samples was read on a spectrophotometer at a wavelength of 450 nm. The sensitivity of the sPD-1 ELISA was 1.14 pg/ml. There was no cross-reactivity or interference detected between natural and recombinant human PD-1.

### Statistical analysis

Statistical analysis of data was performed using PRISM (version 5,GraphPad Software, La Jolla, CA, USA) and/or SPSS for Windows (version 18.0,SPSS Inc., Chicago, IL, USA). Differences between groups were analyzed by Student’s t test. Comparisons of categorical variables were conducted using Pearson chi-square tests. For nonparametric data, results were expressed as median [interquartile range (IQR)] values, and the differences between groups were analyzed by the Mann–Whitney U test. Spearman’s correlation coefficient was applied to detect the correlation between two groups. Univariate logistic regression analysis was performed to determine the factors associated with the presence of ILD. Multivariate logistic regression analysis was performed by including the confounding factors that were found to be significantly associated with the univariate analyses. The factors were selected in a stepwise manner owing to the small number of events in the logistic model. The receiver operating characteristic (ROC) curve was used to determine the best cut off values and validity of certain variables. Data are presented as mean ± standard deviation (SD). A *P* value of less than 0.05 was considered statistically significant.

## Results

### Clinical characteristics of patients with RA-ILD

One hundred and thirty-two individuals were included in this study, including 58 with RA-ILD, 29 with RA but not ILD (RA-non-ILD), and 45 healthy controls (HC). Female-to-male ratios in RA-ILD and RA-non-ILD groups were 1.6:1 and 6.25:1, respectively (*P* = 0.02). There was a significant difference in smoking exposure between the RA-ILD and RA-non-ILD groups (25.9% vs 3.4%, *P* = 0.011) (Table 2).

**Table 2:** Clinical characteristics of rheumatoid arthritis (RA) patients with or without interstitial lung disease (ILD)

	RA-ILD (n = 58)	RA-non-ILD (n = 29)	*P* value
Age (years, mean ± SD)	65.7 ± 9.4	61.8 ± 9.2	0.096
Female, (n,%)	36 (62.1)	25 (86.2)	0.02*
Disease duration (months), median, IQR	60 (19.5,120)	72 (5.5,46.5)	0.772
Smoker, (n, %)	15(25.9)	1(3.4)	0.011*
Serum immunology			
Anti-CCP positive, n, %)	54 (93.1)	21 (75.0)	0.008*
Anti-CCP titer(RU/ml), mean ± SD	696.9 ± 531.4	429.3 ± 555.8	0.004*
RF positive, (n, %)	51(87.9)	22(75.9)	0.148
RF titer (IU/ml), mean ± SD	615.8 ± 1186.5	213.8 ± 194.9	0.289
ANA positive, (n, %)	29 (50.0)	14 (48.3)	0.879
Anti-ds-DNA, (n, %)	0 (0.0)	1 (3.4)	0.155
Anti-SSA, (n, %)	7 (12.1)	1 (3.4)	0.197
Anti-Ro52, (n, %)	9 (15.5)	4 (13.8)	0.832
Anti-PM-Scl, (n, %)	0 (0.0)	1(3.4)	0.155
Anti-RNP, (n, %)	1 (1.7)	0(3.4)	0.481
Anti-centromere, (n, %)	2 (3.4)	0 (0.0)	0.312
Anti-phospholipid, (n, %)	4 (6.9)	2 (6.9)	> 0.99
ANCA, (n, %)	4 (6.9)	5 (17.2)	0.135
Disease activity and treatment			
CRP (mg/dl), mean ± SD	32.8 ± 44.3	39.2 ± 35.5	0.109
ESR (mm/h), mean ± SD	54.2 ± 34.3	47.1 ± 30.6	0.716
IgG (g/L), mean ± SD	13.67 ± 4.42	13.31 ± 3.06	0.642
Ferritin (ng/L), mean ± SD	213.4 ± 128.7	188.2 ± 180.5	0.341
DAS28-ESR, mean ± SD	4.8 ± 1.5	4.7 ± 1.2	0.739
Sharp score, mean ± SD	18.4 ± 22.2	29.8 ± 24.7	0.099
Use of biologics, (n, %)	11 (18.9)	0 (0)	0.012*
Use of MTX, (n, %)	10 (17.2)	18 (62.1)	< 0.0001*
Use of GCs, (n, %)	45 (77.6)	16 (55.2)	0.03*
Dose of GCs (mg/day), mean ± SD	13.9 ± 12.8	7.6 ± 7.6	0.024*

*sPD-1* soluble programmed death molecule-1, *anti-CCP* anticitrullinated peptide antibody, *RF* rheumatoid factor, *ANA* antinuclear antibody, *Anti-ds-DNA* Anti-double-stranded DNA, *ANCA* anti-neutrophil cytoplasmic antibody, *CRP* C-reactive protein, *ESR* erythrocyte sedimentation rate, *IgG* immunoglobulin G, *DAS28* disease activity score using 28 joint counts, *MTX* methotrexate, *GCs* glucocorticoids (in prednisolone equivalent)

–: NA; *P < 0.05

The RA-ILD group had a higher positive rate and increased level of anti-CCP (93.1% vs 75.0%, *P* = 0.008 and 696.9 ± 531.4 vs 429.3 ± 555.8, *P* = 0.004, respectively), but there were no significant differences for RF, anti-nuclear, anti-ds-DNA, anti-Sjögren's-syndrome-related antigen A, and anti-Ro52 antibody levels between the two disease subgroups. Additionally, no significant differences were observed in the “Disease Activity Score-28 for Rheumatoid Arthritis with ESR”, CRP, ferritin, and Sharp scores. Patients in the RA-ILD group had more traditional medical therapy, including use of biologics and glucocorticoids, than those in the RA-non-ILD group (18.9% vs 0.0%, *P* = 0.012 and 77.6% vs 55.2%, *P* = 0.03). Patients with RA-ILD received a higher mean dosage of glucocorticoid treatment (13.9 ± 12.8 mg/L vs 7.6 ± 7.6 mg/L, *P* = 0.024). However, the percentage of methotrexate (MTX) used was less in the RA-ILD group than RA-non-ILD group (17.2% vs 62.1%, *P* < 0.0001) (Table 2).

### Increased serum levels of sPD-1 in patients with RA-ILD

**Fig. 1:**
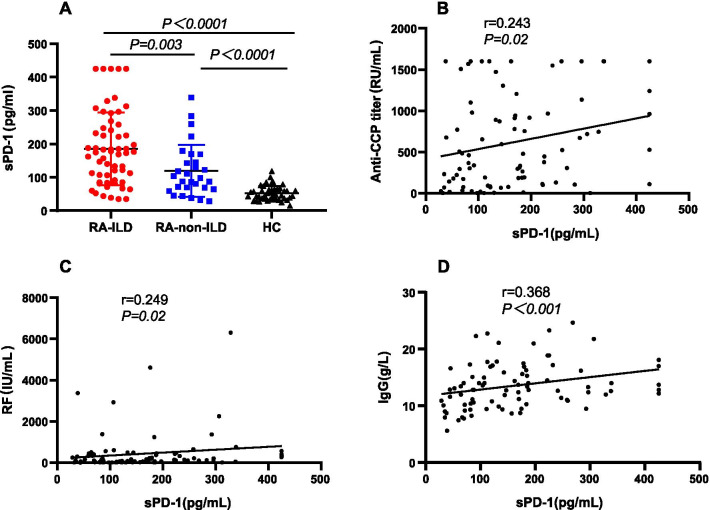
Concentrations of sPD-1 in patients with rheumatoid arthritis (RA) and Healthy controls (HC) (**A**) and their relationship between anti-CCP (**B**), rheumatoid factor (RF) (**C**) and IgG (**D**)

Levels of serum sPD-1 were significantly higher in the RA-ILD group than in the RA-non-ILD group (185.1 ± 109.0 pg/ml vs 119.1 ± 77.5 pg/ml, *P* = 0.003, Fig. 1A) and healthy controls (185.1 ± 109.0 pg/ml vs 52.1 ± 21.7 pg/ml, *P* < 0.0001, Fig. 1A). There were significant correlations between serum sPD-1 and anti-CCP or RF levels (*P* = 0.02, r = 0.243, Fig. 1B, and *P* = 0.02, r = 0.249, Fig. 1C, respectively). Serum sPD-1 was also positively correlated with serum IgG levels (*P* < 0.001, r = 0.368, Fig. 1D), but not with other laboratory parameters, including CRP, ESR, and ferritin.

### Serum sPD-L1 is associated with the occurrence of ILD in RA

**Fig. 2:**
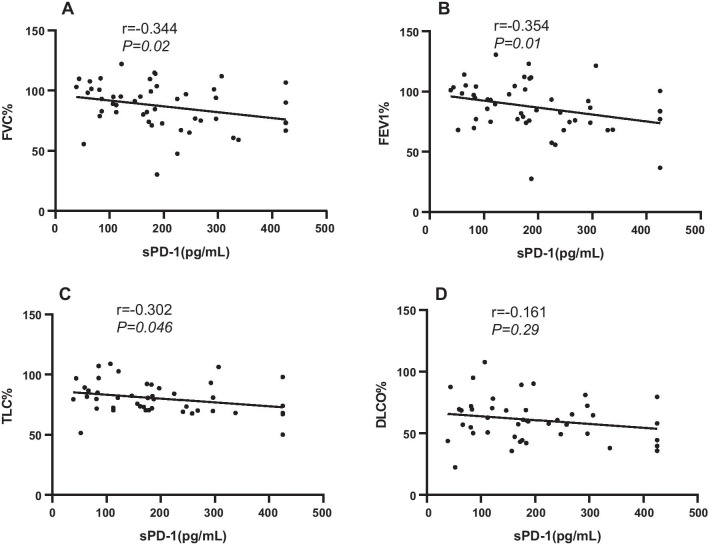
Correlation between the serum levels of sPD-1 with the forced vital capacity percent predicted values (FVC%, **A**), Forced expiratory volume in one second predicted values (FEV1%, **B**), Total lung capacity predicted values (TLC%, **C**) and diffusing capacity for carbon monoxide percent predicted values (DLCO%, **D**) in RA-ILD

To further evaluate the relationship between sPD-1 and RA-ILD, the correlations between sPD-1 and lung function indexes in RA-ILD were analyzed. The results showed that sPD-1 was negatively correlated with FVC% (*P* = 0.02, r =  − 0.344, Fig. 2A), FEV1% (*P* = 0.01, r =  − 0.354, Fig. 2B) and TLC% (*P* = 0.046, r =  − 0.302, Fig. 2C), but there was no association between sPD-1 levels and HRCT score and DLCO% (*P* = 0.29, r =  − 0.161, Fig. 2D).

### Serum sPD-1 is a risk factor for the occurrence of ILD in RA

**Fig. 3:**
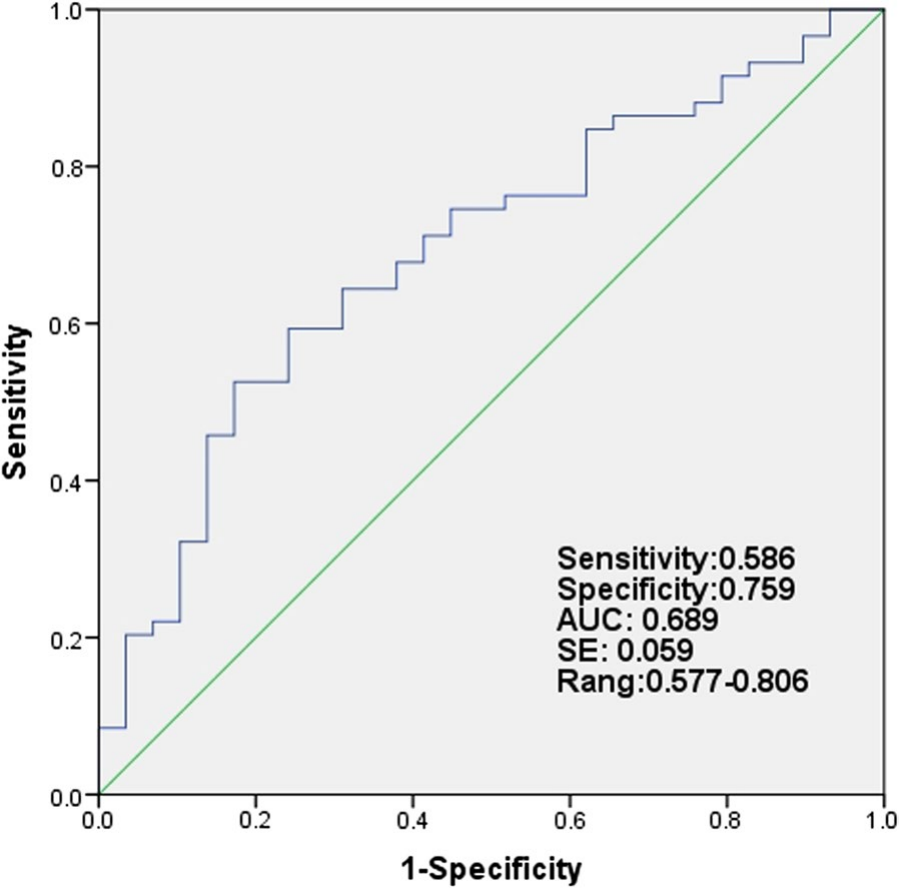
Area under the curve (AUC) and sensitivity and specificity of sPD-1 levels in RA patients using receiver operating characteristics curve analysis for the detection of RA with ILD

Univariate and multivariate logistic regression analyses were performed to determine the related factors of ILD in patients with RA. The univariate analysis showed that sPD-1, female sex, smoking history, anti-CCP positivity, use of MTX and glucocorticoid, and the dose of glucocorticoid were all factors associated with RA-ILD. In multivariate logistic regression, after adjusting the confounding factors, serum sPD-1 was found to be an independent risk factor for the presence of ILD (*P* = 0.020, Table 3). Finally, ROC curve analysis showed that the area under the curve of sPD-1 expression was 0.689, and the area under the ROC curve was greater than 0.5 (*P* < 0.05). The cutoff value of serum sPD-1 was 145.4 pg/ml, with a sensitivity of 0.586 and specificity of 0.759 (Fig. 3).

**Table 3:** Univariate and multivariate logistic regression analysis of related factors of interstitial lung disease (ILD) in patients with rheumatoid arthritis (RA)

	Univariate logistic analysis		Multivariate logistic analysis	
	OR (95% CI)	*P* value	OR (95% CI)	*P* value
sPD-1	1.00 (1.00–1.01)	0.008*	1.012 (1.002–1.023)	0.020*
Age	1.05 (0.99–1.10)	0.073		
Female	0.27 (0.08–0.88)	0.029*	0.135 (0.005–3.981)	0.246
Disease duration	1.00 (0.95–1.06)	0.977		
Smoking history	9.77 (1.22–78.14)	0.032*	2.35 (0.042–130.83)	0.677
DAS28-ESR	1.05 (0.77–1.44)	0.768		
Sharp score	0.86 (0.69–1.07)	0.171		
Anti-CCP positive	8.75 (1.69–45.44)	0.029*	10.601 (0.602–186.681)	0.107
Anti-CCP titer	1.00 (1.00–1.01)	0.014*	1.001 (0.999–1.003)	0.587
RF positive	1.94 (0.59–6.41)	0.278		
RF titer	1.00 (0.99–1.01)	0.175		
Ferritin	1.00 (0.99–1.01)	0.641		
Use of biologics	5.71 (0.70–47.01)	0.105		
Use of MTX	0.13 (0.05–0.34)	< 0.0001*	0.052 (0.009–0.292)	0.0008*
Use of GCs	2.61 (1.01–6.73)	0.047*	6.517 (0.588–72.206)	0.127
Dose of GCs	1.06 (1.01–1.12)	0.021*	1.024 (0.927–1.131)	0.637

*sPD-1* soluble programmed death molecule-1, *DAS28* disease activity score using 28 joint counts, *anti-CCP* anticitrullinated peptide antibody, *RF* rheumatoid factor, *MTX* methotrexate, *GCs* glucocorticoids (in prednisolone equivalent)

–: NA; *P < 0.05

### Serum sPD-1 levels may provide a disease marker to predict RA with ILD

According to the cutoff value of serum sPD-1 obtained by the ROC curve, patients with RA-ILD were divided into high-level and low-level groups. The clinical characteristics of the two groups were compared. FVC% was significantly lower in the high-level group than in the low-level group (82.5 ± 20.1 vs 94.8 ± 15.2, *P* = 0.022). We found that the number of patients with cough, dyspnea, and chest tightness in the high-level group was higher than that in the low-level group, and the DLCO% data were also lower than that in the low-level group (Table 4), but without statistical significance.

**Table 4:** Comparison of clinical characteristics of RA-ILD patients with different levels of sPD-1

	sPD-1		*P* value
	High-level group n = 34	Low-level group n = 24	
Age (years, mean ± SD)	66.0 ± 9.7	65.2 ± 9.0	0.689
Female, (n, %)	22 (64.7)	15 (62.5)	0.863
Disease duration (months), median, IQR	60 (12, 141)	60 (36, 120)	0.548
Smoking history, (n, %)	9 (26.5)	5 (20.8)	0.621
RF titer (IU/ml), mean ± SD	538.8 ± 1151.9	542.8 ± 928.1	0.989
Anti-CCP positive, mean ± SD	32 (94.1)	22 (91.7)	0.717
ESR (mm/h), mean ± SD	48.9 ± 34.1	53.5 ± 31.4	0.60
CRP (mg/dl), mean ± SD	36.0 ± 51.1	29.3 ± 33.9	0.549
DAS28-ESR, mean ± SD	4.5 ± 1.3	5.2 ± 1.7	0.105
HRCT score, mean ± SD	6.2 ± 3.1	5.3 ± 3.7	0.319
CT-UIP (n, %)	23(67.6)	11(45.8)	0.096
FVC%, mean ± SD	82.5 ± 20.1	94.8 ± 15.2	0.022*
DLCO%, mean ± SD	58.4 ± 15.5	66.2 ± 20.6	0.201
Cough (n, %)	21 (61.8)	10 (41.7)	0.131
Dyspnea (n, %)	6 (17.6)	3 (12.5)	0.722
Chest tightness (n, %)	14 (41.2)	6 (25.0)	0.202
Use of GCs (n, %)	23 (67.6)	21 (87.5)	0.121
Use of MTX (n, %)	6 (17.6)	3 (12.5)	0.722

*sPD-1* soluble programmed death molecule-1, *ESR* erythrocyte sedimentation rate, *CRP* C-reactive protein, *DAS28* disease activity score using 28 joint counts, *HRCT* high resolution computerized tomography, *UIP* usual interstitial pneumonitis, *RF* rheumatoid factor, *anti-CCP* anticitrullinated peptide antibody, *MTX* methotrexate, *GCs* glucocorticoids (in prednisolone equivalent)

## Discussion

This is the first study investigating circulating levels of sPD-1 in patients with RA-ILD. The current findings revealed significantly higher sPD-1 levels in patients with RA-ILD compared with patients exhibiting RA without ILD and healthy controls.

Interstitial lung disease is the most common pulmonary manifestation of RA, and has poor prognosis. Approximately 10–14% of patients with RA develop clinically significant interstitial lung disease [1, 10]. Additionally, approximately 30% of patients with RA are diagnosed with subclinical ILD if examined by a HRCT scan [2, 11]. The risk of death for patients with RA-ILD was three times that of non-ILD RA patients [12]. Several multifactor components may assist in the development of ILD, including associated risk factors covering environmental, serological, clinical, genetic, and drug-related components.

Tobacco use has previously been identified as a risk factor for ILD. It has also been shown that a significantly higher proportion of subjects with subclinical RA-ILD are current and former smokers [13], and the incidence of ILD increases as the amount of smoking increases [14]. Other studies have shown that RA-ILD often occurs within 10 years of the onset of RA and the presence of ILD is related to disease duration and RA disease activity [15]; patients with RA-ILD have higher disease activity. Research has shown that the female sex was a protective factor against RA-ILD [16]. In our study, we found that the RA-ILD group had more male participants and smokers than the RA-non-ILD group, which is consistent with previous studies [13, 14, 16]. However, we did not observe any correlation between disease activity and the occurrence of ILD. Because of the limitation of a retrospective study approach, the small sample size and lack of longitudinal data in the current investigation, the impact of disease activity on the development of interstitial pneumonia remains unclear.

Current investigations of biomarkers aim to provide different methods for earlier diagnosis and evaluation of ILD activity and severity. Among several biomarkers, Krebs von den Lungen-6, pulmonary surfactant-associated protein D and matrix metalloproteinase-7 were reported to be useful for predicting idiopathic and connective tissue disease-related ILD [17–19]. In addition, ferritin, lactate dehydrogenase, anti-CCP, and RF have been also reported to be highly expressed in patients with RA-ILD [20–23]. But results from different cohorts were quite variable and controversial. It is generally recognized that anti-citrullinated protein antibody and RF are related to the severity of the disease and extra-articular damage. In the present study, we found that the positive rate of anti-CCP in patients with RA-ILD was nearly 95%; these patients had higher anti-CCP titers compared with patients in the RA non-ILD group, consistent with previous reports [22, 24].

The expression of sPD-1 can be readily detected in peripheral blood [25]. sPD-1 inhibits the PD-1/PD-L signaling pathway by interacting with PD-Ls, and therefore promotes the activation of T cells [26]. Previous studies have demonstrated that the expression of sPD‑1 was elevated in patients with RA and was correlated with the disease activity [27, 28]. However, there is no relevant research on whether it is related to RA-ILD. Our study showed that serum sPD-1 levels were significantly elevated in patients with RA-ILD compared with healthy control subjects and RA-non-ILD patients. Further analysis showed that serum sPD-1 was negatively correlated with lung function indexes, including FVC% and FEV1%. Multivariate regression analysis showed that PD-1 was a risk factor in RA-ILD. In addition, the ROC curve exhibited discriminating capacity and the optimal threshold, according to the ROC curve, for serum sPD-1 was 145.4 pg/ml. Therefore, sPD-1 may be a new biomarker to predict the occurrence of ILD. Unfortunately, we did not observe an association of sPD-1 with the severity of ILD in RA.

The pathogenesis of RA-ILD is still unclear. T lymphocytes are considered important for the pathogenesis of RA. Many studies have shown that the imbalance of lymphocyte subsets plays an important role in the occurrence and development of ILD [29]. Previous studies reported that RA-ILD and IPF have overlapping pathogenesis, providing a new approach to study the pathogenesis of RA-ILD [30]. Wang et al. [31] found that PD-1 positive CD8^+^ T cells were significantly increased in lung tissue samples of patients with IPF, and the investigators speculated that abnormally activated T lymphocytes, particularly CD8^+^T lymphocytes, may be the main cell subsets inducing immune damage in pulmonary fibrosis. sPD-1 is expressed by CD4^+^ and CD8^+^ T lymphocytes stimulated by proinflammatory cytokines [25]. sPD-1 may be a facilitating factor for pulmonary fibrosis. But this hypothesis may require further investigation.

Leflunomide, MTX, and TNF-α antagonists are commonly used in the treatment of RA. These treatments have been considered beneficial for arthritis control, but include some side effects, such as interstitial pneumonia [10]. About 0.43% of MTX-treated patients exhibited a rare side effect of allergic pneumonia [32]. This type of organ-specific allergic reaction has received considerable clinical attention and is believed to be associated with the incidence rate or exacerbation of RA associated with ILD. This side effect has become one of the reasons clinicians rarely prescribe MTX for patients with pulmonary diseases. In our study, the number of patients prescribed MTX was significantly lower in the RA-ILD group than that in RA-non-ILD group. However, recent studies suggest that MTX treatment is not associated with an increased risk of RA-ILD diagnosis. Conversely, MTX treatment may be a protective factor for ILD in RA [33]. It has also been reported that the use of biological agents, especially the use of tumor necrosis factor (TNF-α) and glucocorticoids, is related to the occurrence of ILD [34, 35]. Our study found that the RA-ILD group had more exposure to TNF-α and glucocorticoids than the RA-non-ILD group. By combining these treatments, patients with RA-ILD may experience increased inflammation.

## Conclusion

In conclusion, increased sPD-1 may be an important biomarker for predicting the occurrence of interstitial pneumonia in patients with RA. Our findings may help to elucidate the implications of high sPD-1 in patients with RA with ILD, and provide new possibilities for managing RA-ILD, as well as important insight into the pathogenesis of ILD.

## Supplementary Information

**Additional file 1**. The details characteristics of rheumatoid arthritis (RA) patients with or without interstitial lung disease (ILD).

## Data Availability

Datasets used in this analysis are available from the corresponding author upon request. Li X, Lichun J, Yan D, Jing X, sPD-1 as predictor of interstitial lung disease in rheumatoid arthritis data sets. figshare.2021. http://dx.doi.org/10.6084/m9.figshare.14754312.

## Abbreviations

sPD-1: Soluble programmed death molecule 1
ELISA: Enzyme-linked immunosorbent assay
IPF: Idiopathic pulmonary fibrosis
RA-ILD: Rheumatoid arthritis-related interstitial lung disease
FVC: Forced vital capacity
FEV1: Forced expiratory volume in one second
DLCO: Carbon monoxide diffusion rate
HRCT: High-resolution computed tomography
TNF-α: Tumor necrosis factor alpha
