# Supplementary material for: Soluble programmed death molecule 1 (sPD-1) as a predictor of interstitial lung disease in rheumatoid arthritis
Source: BMC Immunol. 2021 Oct 15;22:69. doi: 10.1186/s12865-021-00460-6 (PMC8518160; doi:10.1186/s12865-021-00460-6)
Supplement: Supplementary file 1 — Additional file 1. The details characteristics of rheumatoid arthritis (RA) patients with or without interstitial lung disease (ILD). [file 12865_2021_460_MOESM1_ESM.docx]

Table S1: The details characteristics of rheumatoid arthritis (RA) patients with interstitial lung disease (ILD).

| Number | Gender  （M/F） | Ages  （yeas） | Duration  （months） | Smoking history | DAS28-ESR | Anti-CCP titer | RF titer | CRP  (mg/L) | biologics | MTX | Other Dmards | Dose of GCs (mg/day) |
| --- | --- | --- | --- | --- | --- | --- | --- | --- | --- | --- | --- | --- |
| 1 | F | 49 | 216 | No | 5.9 | ＞1600 | 52.2 | 0.7 | No | No | TW, SSZ | No |
| 2 | F | 72 | 240 | No | 7 | 302.98 | 126 | 20.6 | No | Yes | LEF, SSZ, lguratimod | 2.5 |
| 3 | F | 67 | 228 | No | 3.4 | 1205.57 | 38.3 | 3.6 | No | No | TW | 10 |
| 4 | F | 60 | 18 | No | 7.3 | 676.68 | 3375.5 | 69.5 | AdalimumabTocilizumab | No | TW, LEF, AZA | 50 |
| 5 | M | 63 | 12 | Yes | 6.5 | 507.66 | 459 | 0.39 | No | No | TW, lguratimod | 15 |
| 6 | F | 64 | 360 | No | 5.4 | 367.8 | ＜9.69 | 5.13 | No | Yes | HCQ | 15 |
| 7 | M | 73 | 12 | No | 4.75 | 311.7 | 188 | 6.8 | No | No | TW | No |
| 8 | F | 65 | 6 | No | 6.3 | 779.9 | 163 | 119.8 | Tocilizumab | No | TW, SSZ | 17.5 |
| 9 | F | 51 | 360 | No | 4.1 | 130.4 | 103 | 9 | No | No | TW, LEF | 25 |
| 10 | M | 62 | 36 | Yes | 6.4 | 184.8 | 4610 | 7.9 | Etanercept | Yes | LEF, TW | 15 |
| 11 | M | 78 | 12 | No | 2.9 | 179.6 | 153 | 38.6 | Etanercept | No | TW | 30 |
| 12 | F | 48 | 48 | No | 5.6 | ＞1600 | 650 | 5.1 | No | No | TW | 15 |
| 13 | F | 66 | 36 | No | 6.9 | 770.4 | 443 | 122.6 | No | No | TW, LEF | 30 |
| 14 | M | 60 | 96 | Yes | 4.44 | 462 | ＜12.5 | 5.7 | No | No | LEF, SSZ, lguratimod | 15 |
| 15 | F | 73 | 12 | No | 2.4 | 85.2 | ＜12.5 | 9.45 | No | No | TW | No |
| 16 | F | 66 | 12 | No | 3.4 | 1240.1 | 566 | 6.78 | No | No | TW, MMF | 50 |
| 17 | M | 64 | 13.2 | Yes | 5.5 | 192 | 41 | 24.4 | No | No | TW, HCQ, lguratimod | No |
| 18 | M | 67 | 60 | Yes | 2.7 | ＞1600 | 137 | 3.9 | Etanercept | No | HCQ, lguratimod | No |
| 19 | F | 74 | 72 | No | 6.12 | ＞1600 | 24.4 | 10.3 | No | No | SSZ, lguratimod | 15 |
| 20 | M | 80 | 360 | Yes | 5.7 | ＞1600 | 411 | 57.2 | Etanercept | No | TW | No |
| 21 | F | 71 | 300 | No | 3.7 | 754.9 | 451 | 27.8 | No | No | TW | 10 |
| 22 | F | 76 | 108 | No | 4.5 | 188.3 | ＜12.5 | 4 | No | No | TW | 10 |
| 23 | F | 74 | 360 | No | 5.1 | ＞1600 | 57.2 | 6 | No | Yes | TW, SSZ | 20 |
| 24 | F | 67 | 108 | No | 3.7 | 446.1 | 473 | 64.8 | No | No | TW, LEF | 15 |
| 25 | M | 70 | 36 | Yes | 6.18 | 745.2 | 6310 | 182.3 | No | No | TW, SSZ, HCQ, lguratimod | 17.5 |
| 26 | F | 79 | 12 | No | 2.73 | 478.7 | 73.9 | 1 | Tocilizumab | No | TE, LEF | No |
| 27 | M | 78 | 12 | Yes | 4.2 | 527.2 | 342 | 22.2 | No | Yes | TW | 15 |
| 28 | M | 68 | 96 | Yes | 6.3 | ＞1600 | 2930 | 5 | No | No | TW | No |
| 29 | M | 78 | 132 | No | 6.1 | 193.8 | 370 | 52.47 | No | No | TW | 15 |
| 30 | M | 62 | 96 | No | 6 | 378 | 110 | 79.4 | No | No | TW | 20 |
| 31 | F | 60 | 60 | No | 2.3 | 966.52 | 321.8 | 58.9 | No | No | TW, lguratimod | No |
| 32 | F | 50 | 108 | No | 5.4 | 916.58 | 62.3 | 2.3 | Etanercept | Yes | LEF, HCQ | No |
| 33 | F | 67 | 240 | No | 3.73 | ＞1600 | 34 | 8.7 | No | No | LEF | 2.5 |
| 34 | F | 54 | 60 | No | 5 | 1135.9 | 209 | 63.74 | Etanercept | Yes | TW, LEF | 50 |
| 35 | F | 81 | 48 | No | 3.26 | 76.2 | 24.8 | 3.7 | Etanercept | No | TW | 15 |
| 36 | F | 63 | 120 | No | 5.1 | 265.2 | 1240 | 2.6 | No | No | TW, LEF, lguratimod | 10 |
| 37 | F | 64 | 60 | No | 4 | 654.2 | 608 | 25.8 | No | No | TW, LEF, lguratimod | 5 |
| 38 | M | 61 | 9 | Yes | 2 | 719.38 | 2250 | 22.76 | No | No | LEF | No |
| 39 | M | 60 | 240 | Yes | 4.5 | 1550.5 | 146 | 1.7 | No | No | TW, SSZ | No |
| 40 | M | 82 | 240 | Yes | 3.2 | 110.3 | 313 | 65.2 | No | No | TW, lguratimod | 10 |
| 41 | M | 47 | 48 | Yes | 3.9 | 1508 | 331.1 | 23.9 | No | No | Tofacitinib, LEF | 5 |
| 42 | F | 55 | 144 | No | 3.8 | 297.43 | 221 | 0.2 | No | No | TW | 30 |
| 43 | F | 57 | 36 | No | 3.2 | 1305.1 | 70.1 | 6.3 | Etanercept | No | No | No |
| 44 | F | 55 | 120 | No | 3.2 | 672 | 1370 | 4.6 | No | Yes | TW, LEF,  SSZ, lguratimod | 10 |
| 45 | F | 75 | 120 | No | 5.9 | 306.69 | 99.3 | 72 | No | No | TW, SSZ | 40 |
| 46 | F | 50 | 60 | No | 7.7 | 1100 | 1380 | 29.8 | No | No | TW, SSZ, LEF | 20 |
| 47 | F | 62 | 24 | No | 3.5 | 962.40 | 266.4 | 5.7 | No | Yes | TW | 15 |
| 48 | M | 53 | 36 | NA | 1.1 |  | 2 | 1.8 | No | No | TW, lguratimod | 30 |
| 49 | M | 71 | 1 | Yes | 5.3 | 9.65 | ＜11.1 | 10.2 | No | No | TW | No |
| 50 | F | 59 | 48 | No | 6 | 338 | 559 | 52.1 | No | No | TW, LEF | 15 |
| 51 | M | 72 | 60 | No | 6.2 | 1472 | 80.3 | 115 | No | No | TW | 15 |
| 52 | F | 73 | 60 | No | 7 | 873 | 156 | 67.2 | No | No | TW, LEF | 20 |
| 53 | F | 59 | 12 | No | 3.3 | 890.66 | 494 | 2.34 | No | No | TW | 10 |
| 54 | F | 77 | 120 | No | 5.9 | 172.4 | 59.7 | 11 | No | No | TW | 15 |
| 55 | F | 75 | 96 | No | 7.5 | 112.5 | 146 | 212.8 | No | No | TW, LEF | 20 |
| 56 | F | 82 | 6 | No | 3.9 | 70.2 | 27.1 | 0.43 | No | Yes | LEF, HCQ | 10 |
| 57 | M | 54 | 144 | No | 5.1 | 232.7 | 96.8 | 18.1 | No | No | TE, LEF | 20 |
| 58 | M | 68 | 36 | Yes | 5.5 | 1.8 | 9.1 | 39.4 | No | No | TW | No |

M means male and F means female. TW, tripterygium wilfordii; SSZ, sulfasalazine; LEF, leflunomide; HCQ, hydroxychloroquine sulfate; AZA, azathioprine.

Table S2: The details characteristics of rheumatoid arthritis (RA) patients without ILD.

| Number | Gender  （M/F） | Ages  （yeas） | Duration  （months） | Smoking history | DAS28-ESR | Anti-CCP titer | RF titer | CRP  (mg/L) | biologics | MTX | Other Dmards | Dose of GCs (mg/day) |
| --- | --- | --- | --- | --- | --- | --- | --- | --- | --- | --- | --- | --- |
| 1 | F | 52 | 204 | No | 6.4 | 106.4 | 116.2 | 97.2 | No | Yes | TW | 15 |
| 2 | F | 73 | 6 | No | 5.7 | 24.5 | ＜9.69 | 12.1 | No | No | TW, LEF | 15 |
| 3 | F | 70 | 24 | No | 4.1 | 19.7 | ＜11.4 | 38 | No | No | TW | No |
| 4 | M | 69 | 60 | No | 5.1 | 66.6 | 20 | 138.1 | No | Yes | TW | No |
| 5 | F | 66 | 192 | No | 5.5 | 34.4 | 106 | 3.46 | No | No | TW | 10 |
| 6 | F | 46 | 12 | No | 4.9 | 74 | 88.7 | 61 | No | No | LEF | No |
| 7 | F | 72 | 240 | No | 5.2 | 910.6 | 167 | 63.4 | No | No | TW, LEF | No |
| 8 | F | 63 | 6 | No | 4.5 | 523.7 | 213 | 10.7 | No | Yes | LEF | No |
| 9 | F | 58 | 12 | No | 5.6 | 595.5 | 489 | 65.2 | No | Yes | TW | 20 |
| 10 | F | 60 | 12 | No | 3.1 | 75.7 | 26.6 | 3.9 | No | No | TW | No |
| 11 | F | 76 | 60 | No | 4 | 102.5 | 426 | 11.4 | No | No | TW | No |
| 12 | F | 54 | 108 | No | 4.8 | 142.1 | 56.1 | 4.8 | No | No | TW, LEF | 15 |
| 13 | F | 68 | 120 | No | 3.7 | 979.5 | 80.1 | 16.9 | No | Yes | TW | 10 |
| 14 | F | 61 | 72 | No | 5.37 | 3 | ＜12.5 | 14.7 | No | Yes | HCQ | 5 |
| 15 | F | 42 | 72 | No | 5.02 | ＞1600 | 757 | 63.8 | No | Yes | LEF | No |
| 16 | F | 60 | 12 | No | 4.6 | ＞1600 | 201 | 4.6 | No | No | TW | No |
| 17 | M | 61 | 12 | No | 1.4 | 2.6 | ＜12.5 | 4.7 | No | Yes | TW | No |
| 18 | F | 52 | 60 | No | 4.7 | 1567.7 | 38.1 | 87.4 | No | No | LEF, SSZ | 15 |
| 19 | F | 67 | 108 | No | 6 | 26.7 | 247 | 32 | No | Yes | HCQ | 20 |
| 20 | F | 47 | 336 | No | 5.9 | 5.1 | ＜12.5 | 54.3 | No | Yes | No | No |
| 21 | F | 66 | 360 | No | 5.6 | 222.6 | 331 | 17.7 | No | Yes | No | No |
| 22 | F | 58 | 12 | No | 5 | 2.6 | ＜12.5 | 107.6 | No | Yes | TW | 15 |
| 23 | F | 67 | 120 | No | 5.6 | 333.4 | 240 | 22.3 | No | Yes | TW | 10 |
| 24 | F | 58 | 120 | No | 4 | 91.7 | 54.4 | 21.1 | No | Yes | TW | 10 |
| 25 | F | 64 | 48 | No | 2.3 | 17.4 | 26.5 | 3 | No | No | TW | 10 |
| 26 | F | 76 | 240 | No | 4.16 | 483.3 | 404 | 28.2 | No | Yes | TW | 15 |
| 27 | M | 67 | 120 | No | 6.66 | ＞1600 | 292 | 54.3 | No | Yes | LEF | 20 |
| 28 | M | 71 | 120 | Yes | 4.35 | 295.5 | 504 | 37.5 | No | Yes | TW | 15 |
| 29 | F | 48 | 48 | No | 3.4 | 941.9 | 34.2 | 57.4 | No | Yes | HCQ | No |

M means male and F means female. TW, tripterygium wilfordii; SSZ, sulfasalazine; LEF, leflunomide; HCQ, hydroxychloroquine sulfate; AZA, azathioprine.
